# Health outcomes of sexual and gender minorities after cancer: a systematic review

**DOI:** 10.1186/s13643-021-01707-4

**Published:** 2021-06-21

**Authors:** Mandi L. Pratt-Chapman, Ash B. Alpert, Daniel A. Castillo

**Affiliations:** 1 The George Washington University, School of Medicine and Health Sciences, Washington, DC USA; 2 The GW Cancer Center, The George Washington University, 2600 Virginia Avenue, Suite #324, Washington, DC 20037 USA; 3 Wilmot Cancer Institute, Division of Hematology and Medical Oncology, Department of Medicine, University of Rochester Medical Center, Rochester, USA; 4 Edward G. Miner Library, University of Rochester Medical Center, Rochester, USA

**Keywords:** Sexual and gender minorities, LGBTQI, Sexual orientation, Gender identity, Cancer survivorship, Patient-reported outcomes, QOL

## Abstract

**Purpose:**

Cancer research on sexual and gender minority (SGM) populations is gaining momentum. The purpose of this systematic review was to examine what is currently known in the research literature regarding patient-reported health outcomes after cancer treatment among SGM populations.

**Methods:**

In March 2021, a medical librarian conducted a systematic keyword search on PubMed, Embase, Scopus, Web of Science, PsycINFO, ClinicalTrials.gov, and the Cochrane Central Register of Controlled Trials. The primary inclusion criterion was assessment of at least one physical, psychosocial, emotional, or functional patient-reported health outcome related to the impacts of cancer diagnosis and/or treatment. Articles that met inclusion criteria were reviewed in their entirety, charted in a Word Table, and assessed for quality. Quality considerations included study design, sampling approach, diversity of sample, measures used, and analytic procedures. Studies were synthesized based on type of cancer study participants experienced.

**Results:**

Sixty-four studies were included in the final analysis: most were quantitative, secondary analyses or cross-sectional studies with convenience samples, and focused on people with a history of breast or prostate cancer. Differences between sexual minority men and women in terms of coping and resilience were noted. Few studies reported on experiences of transgender persons and none reported on experiences of intersex persons.

**Conclusions:**

A growing literature describes the patient-reported health outcomes of SGM people with a history of cancer. This study summarizes important between-group differences among SGM and heterosexual, cisgender counterparts that are critical for clinicians to consider when providing care.

**Implications for cancer survivors:**

Sexual orientation and gender identity are relevant to cancer survivors’ health outcomes. Subgroups of SGM people have differential experiences and outcomes related to cancer and its impacts.

## Background

Lesbian, gay, bisexual, transgender, queer, and/or intersex (LGBTQI) populations, also known as sexual and gender minorities (SGM), have been largely ignored in research until recently. While it is likely that these populations have been included in previous research, lack of data collection about sexual orientation and gender identity and lack of prioritizing the health of these populations has led to limited knowledge of their specific needs. Before the National Academies of Sciences 2011 report, *The Health of Lesbian, Gay, Bisexual, and Transgender (LGBT) People: Building a Foundation for Better Understanding*, few studies investigated disparities in cancer-related health outcomes based on sexual orientation and no studies investigated the outcomes of gender minority people [1]. In 2016, the National Institutes of Health (NIH) opened a new office dedicated to SGM health research, designating SGM people a minority population [2]. In 2017, the American Society of Clinical Oncology issued a call to action to reduce cancer health disparities for SGM populations [3].

However, most oncology practitioners have not been trained to address the needs of SGM people, and most cancer centers have yet to institute explicit policies or routine practices to collect sexual orientation and gender-identity data in the electronic medical record, use gender-neutral language on forms, provide SGM-specific support services, and/or require SGM cultural humility training for all staff [4]. Lack of training on the clinical and psychosocial needs of SGM patients perpetuates a system in which patients have to teach their clinicians about how to care for them, resulting in suboptimal care and potentially perpetuating stigmatizing behaviors of clinicians [5, 6]. Fortunately, cancer research on SGM patients has started to gain momentum. This review aimed to synthesize what is currently known about patient-reported health outcomes of SGM people after definitive cancer treatment to inform clinical practice and identify gaps in the literature to guide future research.

### Notes on terminology

In this manuscript and in the review conducted, we used “SGM” as a term meant to encompass diverse people whose gender differs from their sex-assigned-at-birth and/or are not heterosexual. While “SGM” is not a term typically used by LGBTQI people to describe themselves, and the authors do not wish to minoritize LGBTQI people, the authors use this acronym, which has been adopted by the NIH, to be inclusive of a wide range of people, including people who do not identify with the words represented in the acronym “LGBTQI.” If a study is focused on a subgroup within the SGM umbrella, the specific subgroup is referred to rather than the broader term “SGM.” Furthermore, the authors acknowledge that the term “survivor” is not universally embraced. Our use of the term, while imperfect, is for the sake of efficiency of wording. We attempt to, whenever is reasonably efficient, refer to people with a history of cancer rather than a cancer “survivor.”

## Methods

### Protocol

No previous protocol for this study has been published. The search strategy intentionally aimed to cast a wide net before selecting eligible studies for full review. Data were reported following the Preferred Reporting Items for Systematic. Reviews and Meta-Analyses (PRISMA) 2020 guidelines [7, 8].

### Data sources and search strategy

A medical librarian constructed a comprehensive database search in March 2021. The search was conducted using a combination of keywords in the title or abstract and index terms on PubMed and Embase and keyword in the title or abstract only for Scopus, Web of Science (all databases), PsycINFO, ClinicalTrials.gov, and the Cochrane Central Register of Controlled Trials (Cochrane Library). The search strategy included three distinct concepts that were combined using the “AND” Boolean operator: Sexual and Gender Minorities, Cancer, and Survivor. For the complete PubMed strategy, see Appendix 1. Filters were used to exclude conference abstracts, conference papers, and conference reviews from Embase; no other filters were used. EndNote was used to identify duplicates and additional duplicates were manually removed before screening for inclusion began. Reference lists from review articles that were identified through the database search were then hand searched to identify additional articles for possible inclusion.

### Eligibility criteria

Studies were limited to articles published in English that included outcomes of SGM people with a history of a cancer diagnosis. To be included in the review, studies had to investigate and report on at least one physical, psychological, or social patient-reported outcome resulting from impact of a cancer diagnosis and/or treatment: studies with patient experience or satisfaction as the sole endpoint were not included. Commentaries, case studies, abstracts, reviews, dissertations, conference posters, provider-focused trainings and interventions, protocol articles without results, and studies conducted prior to the conclusion of cancer therapy were excluded.

### Critical appraisal

Quality considerations included study design, sampling approach, diversity of sample, measures used, and analytic procedures. Cross-sectional designs, convenience samples, homogenous samples, and non-validated measures were considered limitations. Randomized controlled trials, rigorous qualitative methods, diverse samples, and validated measures were considered strengths.

### Reporting

All included studies were reported in two word tables. Table 1 includes place where study took place, types of SGM subpopulations included, study design, and type of cancer study participants experienced. Appendix 2 reports studies in alphabetical order clustered by age (e.g., AYA) and type of cancer experienced (e.g., breast, colorectal, prostate, various). Studies were synthesized according to experiences of women who have sex with women (WSW), men who have sex with men (MSM), and transgender persons. Given the small number of studies focused on AYA and colorectal cancer survivors, data for these studies were only reported in Appendix 2. Comparisons of SGM subgroups are included in the “Discussion.”

**Table 1:** Characteristics of included studies (*n* = 64)

	Number of studies
Country where study takes place^a^	
Australia	6
Canada	11
Ireland	1
New Zealand	1
Romania	1
Sweden	1
United States of America	53
United Kingdom	2
Reports outcomes of^a^	
AYA SGM	2
SGM broadly	11
MSM	24
WSW	29
Transgender people	5
Intersex people	0
Study design	
Mixed methods	3
Qualitative	13
Quantitative	48
Cancer focus	
Breast	26
Colorectal	2
Prostate	23
Various cancers	13

^a^Not mutually exclusive

## Results

### Study selection

Database searches for peer-reviewed articles focused on health outcomes among SGM persons after definitive treatment for cancer yielded 201 entries in PubMed, 671 in Embase, 344 in Scopus, 279 in Web of Science, 118 in PsycINFO, 12 in ClinicalTrials.gov, and 7 in Cochrane Library’s Central Register of Controlled Trials for a total of 1632 articles. All included articles were required to have SGM people with a history of cancer as a primary focus of the study. Checking for duplicates on EndNote identified 604 duplicates leaving 1028 articles. There were 930 total articles identified for review after manual duplication screen of the EndNote library identified an additional 98 duplicates.

**Fig. 1:**
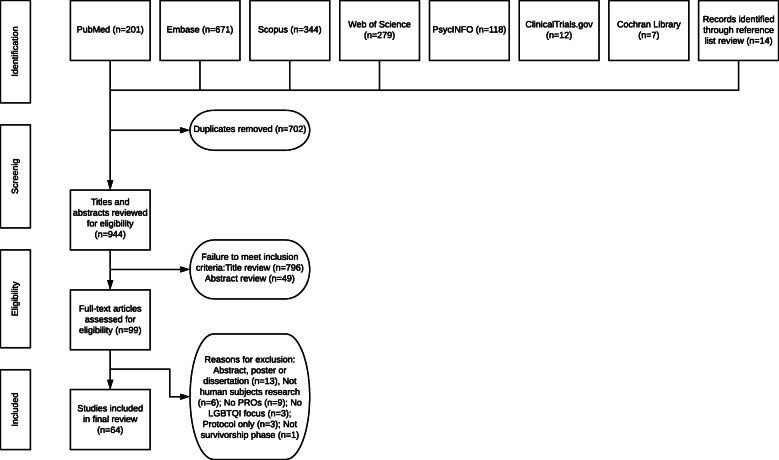
PRISMA diagram of study selection

Authors MPC and AA independently reviewed all titles in Excel for these 930 entries. At the title review stage, 796 articles were eliminated. MPC reviewed all abstracts (*n* = 134), and AA reviewed 10% of the abstracts based on random selection and agreed with MPC on exclusion and inclusion for full-text review. A manual review of reference lists of review articles was conducted to ensure no studies were missed in systematic searches, adding additional 14 articles for abstract review for a total of *n* = 148 abstracts [9]. Forty-nine studies were eliminated at the abstract review stage leaving *n* = 99. MPC and AA each reviewed half of the remaining full texts. Full-text articles were reviewed for compliance with the inclusion criteria; reasons for exclusion of 35 articles after full-text review are provided in Fig. 1. The full-text articles included in the final synthesis were *n* = 64.

### Study characteristics

Most studies were of people with a history of breast or prostate cancer and were focused on disparities based on sexual orientation. Two studies focused on people who were diagnosed with cancer during adolescence and young adulthood (AYA) and two studies focused on people with a history of colorectal cancer. Most studies were conducted in the USA, Australia, and Canada. See Table 1 for general characteristics of included studies.

### Data charting: summary of studies

Appendix 2 was used as a template for data charting. Studies were sorted by the following demographic groups: adolescent and young adults and studies focused on those diagnosed with breast, prostate, or other cancers, respectively. Lead author and year, location, population of interest including any comparison group(s) when relevant, type of study, design, outcomes, and critical appraisal of each study are reported.

### Women who have sex with women

The majority of studies found on SGM people with a history of cancer focused on those who had been diagnosed with breast cancer, mostly comparing lesbians to heterosexual counterparts. Half of the studies on breast cancer were quantitative and analyzed a variety of outcomes from the same two cohorts or subsets of those cohorts [10–17] and [12, 18–20]. Given that half of the analyses were conducted in the same two samples of women, extrapolating findings from these studies on SGM with a history of breast cancer should be done with caution. Nevertheless, studies from these two cohorts combined with additional qualitative studies and two mixed methods studies yielded important insights [21–24].

Participants studied were mostly White, educated, employed, and identified as women. Women who have sex with women (WSW, a term used to be inclusive of lesbian, bisexual, and queer women, and women who do not identify in these ways but partner with women) and heterosexual peers had similar quality of life (QOL) [11, 25] with a few exceptions. WSW with less financial means and those who experienced greater discrimination were more likely to have poorer physical health and increased anxiety and depression [11, 20]. WSW also reported greater stress [26] and less satisfaction with care [24]. In one study, discrimination was associated with anxiety, but resilience and social support buffered this association [26]. Thematic analysis from another study also noted the importance of recognition of partners for psychological wellness, the need for SGM-specific support groups, and the negative impacts of breast cancer treatment on relationships including sexual intimacy [22].

WSW in these studies and their caregivers also showed greater dyadic effects on quality of life compared to heterosexual couples [19]. WSW reported more adaptive coping and improved health behaviors in response to a cancer diagnosis. After cancer diagnoses, WSW with BMI greater than 25 were more likely to lose weight compared with heterosexual counterparts, eliminating a statistically significant pre-diagnosis difference [10]. WSW reported less avoidant coping and anxious preoccupation than heterosexual peers [13, 27] and had similar rates of anxiety and depression [28]. For WSW, having a partner was associated with better sexual function, greater sexual desire, better mental and physical health, and less fear of recurrence compared to heterosexual counterparts [12–14]. WSW also reported that female partners were a singular and valuable source of support and were able to perceive partner distress, manage home and caretaking, and share a life beyond cancer [24]. In addition, WSW reported being less focused on body image, suffered fewer identity issues due to breast cancer and chose not to have reconstruction more often than heterosexual peers [22, 29, 30]. However, WSW reported more challenges with access to care [31] and experienced more physical complications related to mastectomy and radiation than heterosexual peers [15]. Overall, WSW displayed more resilient behaviors than heterosexual peers, although one study indicated there were no between-group differences in resilience based on sexual practices (WSW vs. heterosexual women) [32].

Counter to other studies, one study demonstrated an association between degree of “outness” (defined in the study as the number of relationships in which people were open about their identity) and higher distress [26], which may suggest increased experiences of stigmatization when people were open about their identities. While WSW did not perceive they were treated differently based on sexual orientation, 39% of WSW in one study said they were assumed to be heterosexual by their health care team [25, 33]. Whether level of outness is linked to discriminatory experiences has not been explored.

### Men who have sex with men

Studies on people with a history of prostate cancer primarily focused on genitourinary and relationship changes for men who have sex with men (MSM). Overall, MSM reported more genitourinary challenges than heterosexual peers, including worse urinary and bowel function, lack of ejaculation, changes to erectile function, climacturia, pain during anal sex, penile shortening, loss of libido, and less frequency of sexual activity, although one study demonstrated better sexual function in MSM diagnosed with prostate cancer than that reported in the literature [34–37]. MSM with HIV reported more significant detrimental effects of treatment than MSM without HIV [38]. One study showed that MSM had greater sexual dysfunction after bicalutamide monotherapy compared to heterosexual peers [39]. One novel study assessed the discussions between MSM and their clinicians regarding sexual and urinary effects of prostate cancer and the treatments offered and noted that while the most common problems reported were loss of ejaculate (93.8%), erectile difficulties (89.6%), change in sense of orgasm (87.0%), loss of sexual confidence (76.7%), changes to the penis (65.8%), increased pain in receptive anal sex (64.8%), urinary incontinence not related to sex (64.2%), and urinary incontinence during sex (49.2%), only loss of ejaculate, erectile difficulties, and nonsexual urinary problems were commonly discussed by clinicians during prostate cancer treatment. Satisfaction with specific rehabilitation options varied widely [40].

In qualitative studies, people with a history of prostate cancer reported fearing rejection and sexual abstinence after treatment: “Afterward I felt like I would never find another partner again and there was a depression” [41]. Among MSM who were dating or seeking casual sex, disclosure was seen as a challenge: “A lot of people hit on me, but I just dread that part in the conversation where I have to go, ‘Well, just so you know, I’m a survivor of prostate cancer and there’s never going to be any cum” [42]. Erectile dysfunction led to break-ups in some cases: “For a month or so it was going really nicely, but about a month in he stopped in the middle of sex one night and he said, ‘I’m sorry, you’re just not hard enough for me.’ I was really upset because I was developing feelings for him” [42]. Loss of spontaneity was another noted adjustment:

“Everything has to be planned ahead of time. How much are you drinking? How much salt did you take? Did you take Cialis? If you’re on a date, you may want to have 100 milligram Viagra in your pocket. If you have any chance of going home with somebody, if you want to leave and do that, you can’t drink a lot beforehand because you don’t want to pee in the guy’s bed. All the stuff I never used to think of, ever. It was just wham, bam, thank you, man. You were much more free. Now, all the spontaneity is gone, which is a shame” [42].

Several studies reported changes to participants’ sense of identity as gay men, resulting in changes to relationships and worse mental health [36, 42–44]. In one qualitative study, MSM participants describe erectile dysfunction as a persistent problem that is paramount to being “sexually inferior” or “leading to a sense of ‘disqualification’ of the sexual experience” [36]. Another study reported MSM participants feeling unattractive or even disabled [34]. Sexual changes were reported to adversely affect the mental health and identities of MSM. In Ussher et al.’s [36] study, a gay interviewee reported that erectile dysfunction was “the most horrific thing that I’ve ever been through psychologically.” Another respondent indicated decisional regret, preferring to “take my risks with the cancer” if he could go back in time. One MSM interviewee explained his loss of libido as “a profound change in identity” and another said he felt “outside the sexual community” after the change in his sexual function [36]. Two qualitative studies found that renegotiation of exclusivity was one strategy that couples used to cope with physical symptoms and reduced sexual interest of the survivor. Specifically, survivors in this study encouraged partners to obtain sexual satisfaction outside of their relationship [42, 45]. In contrast, some MSM reported more profound intimacy with their partner [36, 42] after cancer diagnosis and treatment.

Several studies demonstrated benefits of MSM’s disclosure of sexual identity to their providers. In one study, MSM who were comfortable disclosing their sexual orientation had greater masculine self-esteem scores, which was linked to greater mental health [46]. Another study demonstrated reduced anxiety and less illness intrusiveness for MSM who had shared their sexual orientation with providers [35].

Studies also highlighted lack of resources and support tailored for MSM [44, 47]. In Ussher et al.’s qualitative study [36], one MSM respondent summarized the issue like this: “Most health care professionals and others working in the prostate cancer field have no understanding of the different ways that prostate cancer can affect gay and bisexual men. Not just sexually, but in the nonsexual side of relationships. It’s as though we’re invisible.” Other MSM described discomfort with a support group that was mostly attended by heterosexual people: “It’s horrifying because there’s this old man talking about sex with the wife. They don’t want to hear about my problem. I didn’t want to hear about theirs. It didn’t work for me” [41]. In the same study, single MSM expressed the need to be extremely independent and not seek out support: “I was alone to recover… I didn’t really want a lot of company. I mean, I’m walking around the house with a catheter tube sticking out of me, it wasn’t really the time” [41]. Other participants noted that they did not want to bother their friends or chose to hire staff to help them rather than seek help from their friends [41].

### Transgender participants

Only seven studies included transgender and/or genderqueer respondents [21, 22, 31, 48–51]. Boehmer et al. [31] reported an analysis of BRFSS data from 2014 to 2018 that indicated that transgender men had a higher likelihood of having a cancer diagnosis than cisgender men as well as poorer physical health and more comorbidities compared to cisgender men and cisgender women. Bryson et al. [48] found that intersectional identities impacted the experiences of transgender cancer survivors. Brown and McElroy [21] described health care providers “gender policing” when genderqueer patients made the decision to “go flat” and declined breast reconstruction. These experiences and treatment choices were associated with mixed physical and emotional outcomes. Participants reported being unprepared for early menopause and mental health impacts of hysterectomy. Respondents in another study who identified as “queer,” “questioning,” “genderqueer,” “transgender,” or “other” compared with other SGM people were more likely to report that their current level of support was below average [22]. Kamen et al. [50] and Lisy et al. [51] included transgender respondents in their studies, but the former did not stratify outcomes specific to transgender respondents, and the latter did not decouple gender identity from sexual orientation.

### Critical appraisal

Most studies were either (1) secondary analyses of state-level data where data were available on sexual orientation and gender identity in population surveys or (2) cross-sectional surveys of survivors that could be subject to recall, self-selection, and social desirability bias. Only one study assessed the impact of a therapeutic drug on survivor outcomes [39]. The study was based on binary assumptions about sex and sexual orientation. However, it is singular in demonstrating hormone-based differences in response to cancer pharmacotherapies. A strength among many studies was use of validated measures, although sometimes these had to be adapted to be responsive to the experiences of SGM people. Table 2 catalogs measures used in the included studies.

**Table 2:** Summary of validated scales used in SGM survivorship research

Abbreviation	Scale	Reference
BFS	Benefit Finding Scale	Antoni et al. (2001) [52]
BITS	Breast Impact of Treatment Scale	Frierson, Thiel, & Anderson (2006) [53]
BSI-18	Brief Symptom Inventory-18	Derogatis & Melisaratos (1983) [54]
BSS	Berlin Social Support Scale Key	Schulz & Schwarzer (2003) [55]
CapSURE	Cancer of the Prostate Strategic Urologic Research Endeavor	Lubeck et al. (1996) [56]
CES-D	Center for Epidemiological Studies Depression Scale	Radloff (1977) [57]
CHIS	California Health Interview Survey	UCLA Center for Health Policy Research (2012) [58]
CSFQ-M	Changes in Sexual Functioning Questionnaire for Men	Keller, McGarvey, & Clayton (2006) [59]
DAS	Dyadic Assessment Scale	Spanier (1976) [60]
DS-II	Demoralization Scale II	Robinson et al. (2016) [61]
DSC	Dyadic Sexual Communication Scale	Clark et al. (2003) [62]
DSQ	Dyadic Support Questionnaire	Vinokur & Vanryn (1993) [63]
EPIC	Expanded Prostate Cancer Index Composite	Wei et al., (2000) [64]
FACT	Functional Assessment of Cancer Therapy- Prostate	Esper, Mo, & Choadak (1997) [65]
FSFI	Female Sexual Function Index	Rosen et al., (2000) [66]
GAD-7	General Anxiety Disorder-7	Spitzer et al. (2006) [67]
HADS	Hospital Anxiety and Depression Scale	Zigmond & Snaith (1983) [68]
IES-6	Impact of Event Scale-6	Thoreson et al. (2010) [69]
IIEF	International Index of Erectile Function	Rosen et al. (1997) [70]
IIRS	Illness Intrusiveness Ratings Scale	Devins et al. (2001) [71]
ISEL-SF	Interpersonal Support Evaluation List	Cohen et al. (1983) [72]
Mini-MAC	Mini-Mental Adjustment to Cancer Scale	Watson et al. (1994) [73]
QLQ-BR23	EORTC Quality of Life Scale	Aaronson et al. (1993) [74]
MAX-PC	Memorial Anxiety Scale for Prostate Cancer	Roth et al. (2003) [75]
MBSRQ	Multidimensional Body-Self Relations Questionnaire	Cash (2000) [76]
MOSS-SS	Medical Outcomes Study Social Support Survey	Moser et al. (2012) [77]
MSHQ	Male Sexual Health Questionnaire Short Form	Rosen et al. (2007) [78]
MSPSS	Multidimensional Scale of Perceived Social Support	Zimet et al. (1988) [79]
NHANES	National Health and Nutrition Examination Survey	Centers for Disease Control and Prevention (n.d.) [80]
PrCQOL	Prostate Cancer-Related Quality of Life Scales	Clark et al. (2003) [62]
PHQ-8	Patient Health Questionnaire	Kroenke et al. (2009) [81]
PSS	Perceived Stress Scale	Cohen & Wills (1985) [82]
QOL-CSV	Quality of Life-Cancer Survivors	Ferrell, Hassey, & Dow (1997) [83]
RS-14	Resilience Scale	Wagnild & Young, (1993) [84]
RQ	Relationship Questionnaire	Bartholomew & Hororwitz, (1991) [85]
SCNS-SF34	Supportive Care Needs Survey-34	Boyes, Girgis, & Lecathelinais (2009) [86]
SF-12	Medical Outcomes Short Form-12	Ware, Kosinski, & Keller (1996) [87]
SF-36	Medical Outcomes Short Form 36	Ware, Kosinski, & Keller (1994) [88]
STAI	State-Trait Anxiety Inventory	Spielberger, Gorsuch, & Lushene (1968) [89]
TPS	Trust in Physician Scale	Anderson & Dedrick (1990) [90]

## Discussion

### Differences among SGM populations

Most of the studies reviewed were focused on breast cancer for WSW or prostate cancer for MSM. Several studies that synthesized outcomes for people with a history of various cancers indicated worse physical outcomes for SGM compared to heterosexual, cisgender counterparts [91]. However, studies identified no mental health differences between WSW with cancer compared to heterosexual peers. In contrast, a number of studies demonstrated poorer mental health and increased relationship difficulties for MSM with cancer compared to heterosexual peers [92]. In two separate studies, WSW reported lower fear of recurrence while MSM reported greater fear of recurrence compared to heterosexual counterparts [17, 93]. In other studies, MSM were also less likely to be partnered than heterosexual people in contrast to WSW, who were more likely to be partnered [14, 94]. Partner support appeared to buffer negative effects for WSW [13, 17, 29], and partner support was associated with greater reduction in depression [50, 95]. This buffering effect of partnership was not necessarily true for MSM [96]. The heightened dyadic effect of patient-caregiver quality of life shown in Boehmer et al.’s 2020 study [19] highlights the importance of providers including WSW caregivers in cancer treatment discussions.

Additionally, knowledge and competence with SGM health emerged as a critical concern. One study found that SGM who reported their oncologist was not knowledgeable about SGM care reported greater unmet needs and were less likely to disclose their sexual orientation or gender identity to their oncologist [52]. Unmet needs included depression, sadness, cancer-related fears, uncertainty, stress, and sexual dysfunction [52]. Another study found bisexual women who had a history of cancer to be three times more likely to report psychological distress [97]. Bisexual individuals often experience dual discrimination by both mainstream and SGM communities, which may account for this heightened distress. Collectively, these findings support past research that has demonstrated the importance of knowledgeable and unassuming providers in meeting the cancer care needs of SGM persons. For example, a recent study about gender diverse individuals’ satisfaction with care reported discontent with provider assumptions about lesbians wanting breast reconstruction and transgender men wanting hysterectomies; the same study reported that transgender men experienced challenges to male chest reconstruction after breast cancer [98]. Greater clinical and cultural knowledge of SGM concerns and SGM-affirming interventions for concerns are needed.

### Gaps in research

Since the National Academies 2011 report on SGM health, more studies have been published which examine patient-reported outcomes of SGM people with a history of breast or prostate cancers. However, studies exploring the needs and outcomes of sexual minorities with other cancers as well as studies documenting and addressing the needs of gender minorities are severely limited. Only two studies focused on people with a history of colorectal cancer. One study reported financial challenges of queer colorectal cancer survivors; however, there was no comparison group and no other studies with which to compare the sample. Boehmer et al.’s registry-based study of colorectal cancer survivors showed no differences in patient-reported experiences regarding physician communication, nursing care, or coordination of care, but sexual minorities were more likely to categorize their care overall as “excellent” compared to heterosexual counterparts. A major implication of this study is the potential role of resilience among sexual minorities when facing multiple traumatic events across the lifespan. A weakness of the study was the inability to examine differences in MSM and WSW outcomes within studies due to the aggregation of male and female sexual minorities in the literature. A few studies examined outcomes of people diagnosed with diverse cancers, but overall, more research on people surviving a variety of types of cancer is needed to understand differences in health-related outcomes for SGM survivors.

A critical gap exists in studies focusing on transgender, genderqueer, gender diverse, and intersex patient-reported outcomes. Only seven studies mentioned transgender patients [21, 22, 31, 48–51]. Of these, one study mentioned the term “intersex,” yet no intersex people were actually included in the study; in addition, sexual orientation was not reported separately from gender identity, conflating multiple constructs [50]. Of note, intersex people often refer to themselves as female or male rather than intersex. Therefore, intersex individuals may be overlooked within some studies under binary sex categories when intersex status is not assessed.

Another critical gap exists in studies focusing on SGM people of color and SGM people with other intersecting marginalized identities. This work is needed to understand the ways that multiple axes of oppression may affect the outcomes of patients after a cancer diagnosis.

Only two studies focused on AYA people with a history of cancer. Desai et al. [99] found greater likelihood of anxiety among sexual minority AYA survivors compared to heterosexual counterparts. Another qualitative study reported that SGM AYA survivors were less concerned with the possibility of infertility and more open to being non-biological parents than heterosexual peers [100]. More work on AYA cancer survivorship that stratifies experiences and outcomes based on sexual orientation and gender identity is needed.

A strength of many of the studies was the use of validated measures. A list of measures is provided in Table 2 for reference. In some cases, measures used were constructed for heterosexual people and were not relevant to SGM populations. This was particularly true of measures focused on sexual function and outcomes. This limitation of existing measures led researchers to sometimes create or adapt instruments for their studies. Validation of measures focused on SGM sexual outcomes is needed to ensure rigor and reliability of research and to allow for comparisons across studies of SGM survivors.

Finally, only two studies were interventional. Kamen et al. [95] found a dyadic exercise intervention for partners to be more effective in reducing depression than a survivor-only intervention. Anderson et al. [46] found that among elderly gay men who were long-term survivors of AIDS and had another serious medical illness (e.g., cancer), psilocybin-supported group therapy was feasible and appeared to have positive effects. This interventional study was novel as a group-based intervention rather than individual-level intervention coupled with pharmacotherapy for clinically demoralized patients [46]. Interventional research to address poorer physical health among lesbians who have been diagnosed with breast cancer and greater sexual challenges for MSM with prostate cancer are needed. Interventional research that provides early and clear information on fertility preservation is also needed for AYA and other survivors regardless of sexual orientation or gender identity.

### Limitations and strengths of the literature reviewed and of this study

Notably, a limitation of the current literature on SGM people with a history of cancer is lack of diversity of samples, reliance on cross-sectional studies, and lack of interventional studies. Lack of studies in people diagnosed with cancers other than breast or prostate are also significantly lacking. Greater attention to intersectionality, distinctions among SGM subgroups and reporting of data for transgender, gender diverse, and intersex persons are warranted.

This study was limited to articles published through March 2021 focused on SGM people with a history of cancer that reported at least one post-treatment physical, psychological, or social outcome. The study did not include studies that focused only on experiences of care (such as patient satisfaction) unless at least one health-related patient-reported outcome was also included as an endpoint. For a recent review that includes studies of patient satisfaction [101], care decision-making [102, 103], provider training for improved SGM care [104], supportive care needs [104, 105], and other disparities affecting SGM persons [106], see Kent et al. [107]. These studies were not included here, because our focus was on patient-reported outcomes as a result of cancer and treatment rather than satisfaction or care experiences in treatment.

A major strength of this review is its comprehensiveness in summarizing SGM survivorship research to date due to the systematic search methods [8]. This review contributes uniquely to the literature by providing an update to existing reviews [107], focusing on health outcomes as the endpoints of interest, providing a critical appraisal of studies, comparing differences among SGM subgroups (e.g., research findings relevant to sexual minority women v. sexual minority men), and identifying additional gaps in the research literature. Secondary analyses of primary data sets are also included, unlike existing reviews [107]. Finally, a table of measures used in research focused on SGM cancer survivors is included in Table 2. This summary can assist with scale selection and adaptation for future research to aid in comparisons across studies over time.

## Conclusions

This study summarized important between-group differences among SGM people and heterosexual, cisgender counterparts. This review found clear differences in perspectives and health outcomes between WSW and MSM and a lack of reporting regarding whether participants were cisgender or transgender. Thus, researchers should take care to not conflate WSW and MSM when conducting analyses and should ask participants about transgender and gender expansive identities. Gender minority people have been understudied; expanding research in this area will be important to the creation of interventions to improve post-treatment experiences of gender minority people with a history of cancer. Sexual orientation, gender identity, genomic material, hormone balance, and physical anatomy are separate constructs that should not be conflated. Finally, attention to intersectionality within SGM populations is critical as people with multiple intersecting aspects of their identity may have drastically different needs, experiences, and outcomes than those of SGM people who identify with only one marginalized population. Intersectionality was not well-addressed in the extant literature reviewed.

It is paramount that anatomy (including intersex status), sexual orientation, and gender identity be documented in electronic health records and population-based surveys. Until these important variables are systematically recorded and used by clinicians and researchers, SGM research will continue to be restricted to small sample sizes that are not powered to detect subgroup differences. Studies focused on heterosexual, cisgender populations will need to be replicated in convenience samples of SGM patients, which is poor stewardship of research funding dollars. Adding sexual orientation, gender identity, and intersex questions to all studies would be more efficient and provide more robust data to inform clinical care.

Finally, a shift to healthcare that accounts for social determinants of health and intersectionality is critical to effectively address the needs of SGM people with a cancer diagnosis. Clinicians must be trained on how to tailor medical management based on patient values and priorities of care, including considerations for sexual orientation, gender identity, sexual practices, hormone levels, and physical anatomy rather than by monolithic, binary gender markers. Distinctions between sexual orientation and gender identity in research; structured data collection; and clinician training are critical for evidence-based, quality cancer care to improve health outcomes for SGM people. Furthermore, important cultural distinctions within groups that share sexual orientation, gender identity, and/or intersex categories may yield additional insights regarding within-group differences. Accounting for the diversity of lived experiences of SGM people in research design and analysis will help cancer care better address the needs of diverse populations. In sum, a growing literature describes patient-reported health outcomes of SGM people with a history of cancer, but without systematic registries and/or population-based data collection, data will continue to suffer substantial limitations, thereby reducing utility for clinical practice.

## Data Availability

Data sharing is not applicable to this article as no datasets were generated or analyzed during the current study.

## Appendix 1

### PubMed search strategy

("Neoplasms"[Mesh] OR "Cancer*"[tiab] OR "Carcin*"[tiab] OR "Oncolog*"[tiab] OR "Malignan*"[tiab] OR "Neoplas*"[tiab]) AND ("Survivors"[Mesh] OR "Cancer Survivors"[Mesh] OR "Survivorship"[Mesh] OR "Surviv*"[tiab] OR "History of Cancer"[tiab] OR "Cancer History"[tiab]) AND ("Sexual and Gender Minorities"[Mesh] OR "SGM"[Mesh] OR "Gender minorit*"[tiab] OR "Sexual minorit* "[tiab] OR "LGBT*"[tiab] OR "GLBT*"[tiab] OR "Bisex*"[tiab] OR "Gay"[tiab] OR "Gays"[tiab] OR "Lesbian*"[tiab] OR "Pansex*"[tiab] OR "Queer*"[tiab] OR "Asex*"[tiab] OR "Transgender*"[tiab] OR "Transsex*"[tiab] OR "Transex*"[tiab] OR "Intersex*"[tiab] OR "Gender-expansiv*"[tiab] OR "2-spirit*"[tiab] OR "Two-spirit*"[tiab] OR "Non-conform*"[tiab] OR "Non-binar*"[tiab] OR "Same Gender Loving"[tiab] OR "SGL"[tiab] OR "Women Who Partner with Women"[tiab] OR "Men Who Partner with Men"[tiab] OR "Women Who Have Sex with Women"[tiab] OR "Men Who Have Sex with Men"[tiab]).

## Appendix 2

### Summary of studies of patient-reported outcomes from SGM people diagnosed with cancer

**Table:**

Study	Location	Population	Type of study	Study design	Outcomes reported	Critical appraisal
***AYA cancers***						
Desai et al. (2020) [99]	USA	Adolescent and young adult (AYA) cancer survivors (*n* = 1025), 18–40 years old; 64 identifying as sexual minorities.	Quantitative	A cross-sectional study analyses using multivariable logistic regression tested associations between sexual minority status and self-reported anxiety and depression.	Sexual minority AYA survivors had 1.88 higher odds of anxiety compared to heterosexual counterparts, but no statistically significant rates of depression. More social support was associated with less likelihood of depression.	Strengths: Use of validated scales (GAD-7 and PHQ-8); one of very few studies focused on AYA survivors.
Russell et al. (2016) [100]	USA	Adolescent and young adult (AYA) cancer survivors (*n* = 56) including SGM (*n* = 22) and heterosexual (*n* = 34) survivors	Qualitative	AYA survivors were interviewed by telephone; asked about pre- and post-diagnosis thoughts regarding relationships, parenthood, fertility, and how/ if fertility risks were conveyed to them during treatment.	Both SGM and heterosexual survivors reported post-diagnosis dating challenges. Straight survivors had greater fertility concerns (*p* < .05). SGM survivors were more likely to be open to raising a non-biological child or never parenting. Straight survivors were more likely to be unsatisfied with information provided about fertility, but SGM survivors were just as likely to not be informed about potential infertility risks.	Strengths: Only AYA cancer survivorship study known to date that examines differences by sexual orientation; diversity of types of cancer and treatment modalities. Limitations: Small sample size limits subgroup analyses; mostly white sample.
***Breast cancer***						
Bazzi et al. (2018) [32]	USA	BrC survivors: (*n* = 339 heterosexual women, *n* = 201 WSW)	Quantitative	Cross-sectional national survey recruited from Army of Women using multivariable regression with primary outcome as resilience.	Sexual orientation was not associated with resilience, but WSW who were unemployed had less resilience than employed counterparts whereas heterosexual women had no differences based on employment status.	Strengths: Large sample diverse in socioeconomic status, cancer stage, and type of treatment; use of validated scales (ISEL-6, Mini-MAC, RS-14). Limitations: Sample is partially one of convenience, mostly White, and highly educated; self-report data; cross-sectional design.
Boehmer et al. (2011) [10]	USA (Massachusetts Cancer Registry)	Nonmetastatic BrC survivors (*n* = 257 heterosexual women, *n* = 69 WSW)	Quantitative	Multinomial regression with weighting of subpopulations; primary outcome was weight.*	While WSW in the general population were more likely to be overweight and obese, WSW cancer survivors were not statistically more likely to be overweight/ obese than heterosexual counterparts. This finding suggests that WSW may be motivated by cancer to reduce overweight.	Strengths: Recruitment from a population-based registry; diversity of education, socioeconomic status, cancer stage, and treatment modality. Limitations: Data reported from one state; self-report data; potential bias in reporting weight; cross-sectional design.
Boehmer et al. (2012) [28]	USA	Heterosexual women (*n* = 257) and WSW (*n* = 181) diagnosed with nonmetastatic BrC	Quantitative	Using a telephone survey, clinical and demographic characteristics and HADS were assessed. Demographic and clinical factors were compared with *t* tests and chi-square tests and then these characteristics were compared to anxiety and depression assessed via with least squares regression.	The study hypothesis, that WSW who had been diagnosed with BrC had higher rates of anxiety and depression was not confirmed, but sexual orientation was associated with anxiety and depression through interactions with clinical and demographic factors, with younger age and decreased financial means associated with worse anxiety and depression.	Strengths: Use of validated scale (HADS). Limitations: Sample is partially one of convenience, mostly White, and highly educated; self-report data; cross-sectional design.
Boehmer et al. (2012) [10]	USA (Massachusetts Cancer Registry + national convenience sample)	Nonmetastatic BrC survivors (*n* = 257 heterosexual women, *n* = 181 WSW)	Quantitative	Least square regression separately run for physical component and mental component summary scales of the SF-12 on each demographic and clinical characteristic, controlling for sexual orientation.*	Overall, WSW and heterosexual women were comparable in QOL. WSW from the registry were more likely to be White, educated, and employed. Only WSW with low/ middle income had worse physical health than heterosexual counterparts. WSW who experienced more discrimination reported worse physical health.	Strengths: Use of validated scale (SF-12). Limitations: Sample is partially one of convenience, mostly White, and highly educated; self-report data; cross-sectional design.
Boehmer et al. (2012) [11]	USA	Nonmetastatic BrC survivors (*n* = 257 heterosexual women, *n* = 181 WSW)	Quantitative	Least square regression was used for each demographic and clinical characteristic, controlling for sexual orientation.*	WSW appeared more resilient than heterosexual counterparts with some exceptions: unemployed WSW experienced greater anxiety than heterosexual women, and WSW who underwent radiation therapy were more depressed than heterosexual counterparts. WSW reported higher rates of discrimination, which was associated with more depression.	Strengths: Use of a validated measure (HADS); sample size. Limitations: Sample is partially one of convenience, mostly White, and highly educated; self-report data; cross-sectional design; low percentage of variance explained by models.
Boehmer et al. (2012) [29]	USA	Nonmetastatic WSW BrC survivors without recurrence (*n* = 22)	Qualitative	Semi-structured telephone interviews ranging from 30 to 150 min; coding based on grounded theory.	Themes included: 1) BrC is a women’s, not a lesbian, issue; 2) I can manage my identity in the context of BrC; 3) I am better off than my heterosexual counterparts (e.g., less emphasis on body image, empathic female partners)	Strengths: Adaptations to interview guide to maximize neutrality. Limitations: Convenience sample, mostly White, and highly educated; self-report data.
Boehmer et al. (2012) [12]	USA	Nonmetastatic WSW BrC cases and heterosexual controls (*n* = 85 cases, *n* = 85 controls)	Quantitative	Using a conceptual framework for heterosexual BrC survivors, generalized estimating equations identified explanatory factors of sexual function between cases and controls.*	Sexual function was predicted by self-perception of sexual attraction and urogenital symptoms for both WSW and heterosexual women; for partnered women, postmenopausal status and dyadic cohesion was predictive of sexual function; HRQOL was less explanatory for WSW’s sexual function compared to heterosexual women.	Strengths: Case-control design; use of validated scale (SF-12); amount of variance explained by models (nearly half). Limitations: Convenience sample, mostly White, and highly educated; self-report data; use of a sexual measure designed for heterosexual women (FSFI); cross-sectional design.
Boehmer et al. (2013) [13]	USA (Massachusetts Cancer Registry + national convenience sample)	Nonmetastatic BrC survivors (*n* = 257 heterosexual women, *n* = 181 WSW)	Quantitative	Multiple regression models with stepwise variable selection (*p* = .10); model fit reported with *R*^2^ statistics.*	WSW had less cognitive avoidance coping than heterosexual peers. Social support and having a partner were more strongly associated with better mental and physically health, respectively, for WSW v. heterosexual counterparts.	Strengths: Use of validated scales (TPS, ISEL-6, Mini-MAC, BFS); large amount of variance explained in models. Limitations: Sample partially one of convenience, mostly White, and highly educated; cross-sectional design; self-report data.
Boehmer et al. (2013) [14]	USA (Massachusetts Cancer Registry + national convenience sample)	Nonmetastatic WSW BrC survivors (*n* = 161 lesbians, *n* = 19 bisexual women)	Quantitative	Multiple regression models with stepwise variable selection (*p* = .10); fit reported with *R*^2^ statistics.*	Lesbian and bisexual women did not differ in physical or mental health; however, women with female partners fared better than women who were with male partners or unpartnered.	Strengths: Use of validated scales (TPS, Mini-MAC, QLQ-BR23, SF-12); large amount of variance explained in models. Limitations: Small bisexual sample (*n* = 19); sample partially one of convenience, mostly White, and highly educated; cross-sectional design; self-report data.
Boehmer et al. (2013) [15]	USA (Massachusetts Cancer Registry + national convenience sample)	Nonmetastatic BrC survivors (*n* = 257 heterosexual women, *n* = 181 WSW)	Quantitative	Multiple regression (for linear variables) and logistic regression (for dichotomous variables) models with stepwise variable selection (*p* = .10); fit reported with *R*^2^ statistics or pseudo-*R*^2^ statistics.*	WSW generally had lower blood pressure and fewer comorbidities than heterosexual counterparts. However, the impact of mastectomy and radiation in worsening arm symptoms was twice as strong for WSW compared to heterosexual peers. Having health insurance was associated with fewer side effects, an effect three times stronger for WSW v. heterosexual peers.	Strengths: Use of validated scale (QLQ-BR23). Limitations: Sample partially one of convenience, mostly White, and highly educated; cross-sectional design; self-report data.
Boehmer et al. (2014) [16]	USA	Convenience sample of WSW (*n* = 85 with history of BrC, *n* = 85 never-diagnosed)	Quantitative	Case-control study examining sexual frequency, desire, ability to reach orgasm and pain using multiple general linear models or logistic regression for categorical variables.*	Groups did not differ in risk of sexual dysfunction or overall functioning, but cases had lower sexual frequency, less desire and ability to reach orgasm, and more pain during sex.	Strengths: Case-control design. Limitations: Use of a sexual measure designed for heterosexual women (FSFI); cross-sectional design.
Boehmer et al. (2015) [18]	USA	Convenience sample of WSW (*n* = 85 with history of BrC cancer, *n* = 85 never-diagnosed)	Quantitative	Case-control study assessing self-reported physical activity, fruit and vegetable intake, weight, QOL, anxiety and depression using multiple general linear models or logistic regression for categorical variables.*	Groups did not differ in health behaviors, BMI, QOL, anxiety, and depression. Both groups were a majority overweight or obese, around 13-15% reporting depression and 37-45% reporting anxiety. More physical activity correlated with lower weight, less depression, and better mental health in both WSW groups.	Strengths: Case-control design; use of validated scales (HADS, SF-12). Limitations: Cross-sectional design.
Boehmer et al. (2016) [17]	USA	Sample recruited from prior registry-based study plus a sample drawn from the Army of Women (*n* = 167 matched BrC survivor/ caregiver dyads)	Quantitative	Multiple logistic regression on fear of recurrence (FOR) using propensity score matching (*p* < .10). Simultaneous equation models were used to avoid endogeneity, since primary outcomes were patient and caregiver influence on each other’s FOR.	Survivor FOR was explained by years since diagnosis, co-residence with partner, caregiver receiving counseling, survivor ISEL scores, receipt of chemotherapy, and sexual orientation. Caregiver FOR was explained by years since survivor’s diagnosis, caregiver’s discrimination score, caregiver’s social support, survivor’s anti-estrogen therapy, survivor’s comorbidities, and sexual orientation. For both groups, caregiver FOR influenced survivor FOR, but not vice versa. Between groups, WSW survivors and caregivers had less FOR than heterosexual survivors and caregivers.	Strengths: Study design allowed for modeling of causal relationships for FOR. Limitations: Caregiver gender and sexual orientation were not considered; sample lacked racial diversity.
Boehmer et al. (2018) [108]	USA	Sample recruited from prior registry-based study plus a sample drawn from the Army of Women (*n* = 167 matched BrC survivor/ caregiver dyads)	Quantitative	Multiple logistic regression on stress using propensity score matching (*p* < .10). Simultaneous equation models were used to avoid endogeneity, since primary outcomes were patient and caregiver influence on each other’s stress.	WSW survivor and caregiver stress were similar to heterosexual peers; however, WSW dyads showed interdependent stress associations where heterosexual dyads did not.	Strengths: Use of validated scales (ISEL, MSPS, DAS). Limitations: Cross-sectional design.
Boehmer et al. (2020) [19]	USA	Nonmetastatic, non-recurrent BrC survivors of various sexual orientations (*n* = 167)	Quantitative	BrC survivors surveyed by telephone were assessed for QOL; propensity score weighting accounted for differences by sexual orientation in age and length of dyadic relationships; simultaneous equation models assessed dyads.	There were no differences in QOL by sexual orientation 6-7 years post-diagnosis; sexual minority dyads showed greater dependence on partner QOL scores than heterosexual dyads	Strengths: Propensity score weighting; use of simultaneous equation modeling; dyadic assessment; use of validated measures (SF-12, ISEL-SF, MSPSS). Limitations: Cross-sectional design; small comparative heterosexual group.
Brown & McElroy (2018) [21]	USA	WSW diagnosed with BrC	Mixed methods	Purposive and referral sampling were used to recruit WSW diagnosed with BrC to complete an online survey. Bivariate analyses were conducted using cross-tabulations, chi-square statistics, and difference of mean *t* tests to compare those in the sample who identified their sexual orientation as “queer,” “questioning,” or “other” (*n* = 9) and those who identified their gender as “transgender,” “genderqueer,” or “other” (*n* = 11) to the rest of the sample. NVIVO was used for thematic analysis of open-text questions.	Compared to the rest of the sample, those who identified their sexual orientation as “queer,” “questioning,” or “other” or their gender as “transgender,” “genderqueer,” or “other” were more likely to report having bilateral mastectomy without reconstruction, to think that disclosing SOGI to providers affected their care, to use LGBT-specific support groups, and to report that their current level of social support is below average. Thematic analysis revealed themes related to self-disclosure of SOGI to providers, need for recognition and support of partners, need for appropriate social supports for patients and partners, and impact of BrC treatment on intimate relationships	Strengths: Novel study comparing the experiences of lesbian and bisexual cisgender women with BrC to other SGM populations with BrC. Limitations: Study participants who were not cisgender lesbian and bisexual women were identified as “queer” although many did not identify that way. Few people of color were included; predominantly White sample.
Brown & McElroy (2018) [22]	USA	WSW BrC survivors (*n* = 68) ages 18–75	Mixed methods	Purposive and referral sampling were used to recruit WSW BrC survivors to complete an online survey. Bivariate analyses were conducted using cross-tabulations and chi-square tests to determine differences between those electing to choose bilateral mastectomy without reconstruction versus those who did not. NVIVO was used for thematic analysis of open-text questions.	25% of the sample elected to “go flat” or not receive breast reconstruction. “Flattopers” were more likely to identify as genderqueer, be out to their providers, and participate in SGM support groups compared to the rest of the sample. There were not significant between-group differences for the BITS. Qualitative themes from open-text responses included reasons for “going flat,” interactions with health care providers, gender policing/ heterosexism during treatment, and mixed physical and emotional outcomes of treatment choices.	Strengths: This is one of very few studies to report transgender/ genderqueer outcomes of BrC in their own words; use of a previously developed scale (BITS). Limitations: Cross-sectional design; predominantly white sample.
Jabson, Donatelle, & Bowen (2011) [20]	USA	WSW BrC survivors (*n* = 68)	Quantitative	Purposive sampling via known WSW gathering places recruited WSW BrC survivors to participate in an online survey focused on perceived discrimination, social support, stress, and QOL; regression models examined predictive value of independent variables (perceived discrimination, support, stress) on QOL.	Most WSW (92%) reported being treated similar to heterosexual peers. Thirty-nine percent of participants indicated they were perceived as heterosexual by their health care team. Perceived social support and perceived discrimination were statistically significant predictors of better QOL, because perceived heterosexuality was a construct of the discrimination scale and associated with better QOL.	Strengths: use of validated scales (BSS, QOL-CSV, PSS) and adaptation of previous discrimination scale that showed strong reliability (α=.75). Limitations: Predominantly White, educated, insured, partnered, economically stable convenience sample; missing data may skew results toward the null.
Jabson, Donatelle, & Bowen (2011) [33]	USA	BrC survivors (*n* = 143 heterosexual, *n* = 61 WSW women)	Quantitative	Convenience sample of 204 BrC survivors were recruited to an online survey. Means and standard deviations of global QOL and four subscales (physical, psychological, social, and spiritual wellbeing) were compared by sexual orientation (heterosexual v. WSW).	Overall QOL as well as subscales of QOL did not statistically differ between groups.	Strength: Use of validated scale (QOL-CSV). Limitation: Predominantly White, educated, insured, partnered, economically stable convenience sample; missing data may skew results toward the null.
Jabson & Bowen (2014) [25]	USA	BrC survivors (*n* = 143 heterosexual women, *n* = 68 WSW)	Quantitative	Convenience sample of 211 BrC survivors were recruited to an online survey. Means and standard deviations of perceived stress were compared by sexual orientation.	WSW had higher perceived stress than heterosexual peers in regression modeling.	Strength: Use of validated PSS. Limitation: Predominantly White, educated, insured, partnered, economically stable convenience sample; missing data may skew results toward the null.
Kamen et al. (2017) [26]	USA	WSW BrC survivors (*n* = 201) recruited through the Army of Women (*n* = 172 lesbian, *n* = 29 bisexual women).	Quantitative	WSW with stage 0-III BrC completed surveys capturing demographic and clinical factors, minority stress factors, psychosocial resources, and psychological distress factors; linear regression used to examine associations between demographic and clinical characteristics and distress; associations between minority stress, psychological resources, and psychological distress assessed using partial correlations and controlling for demographic and clinical factors associated with distress; structural equation modeling tested direct and indirect effects on distress; statistically significant indirect effects interpreted as mediation.	Discrimination, resilience, and social support were significantly associated with depression after controlling for age, education, income, employment and past chemotherapy. Discrimination, negative identity, resilience, and social support were significantly associated with anxiety. Depression and anxiety were correlated (*r* = .48). Outness and negative identity were significantly positively associated with distress. Resilience and social support were negatively associated with distress. Discrimination had an indirect association with distress mediated by resilience.	Strengths: First study to demonstrate resilience as a positive resource for WSW to buffer the effects of discrimination on distress; use of validated scales (LGB Identity Scale, RS-14, ISEL-SF, HADS). Limitations: Self-report, cross-sectional nature of study; lack of sociodemographic diversity in sample.
Matthews et al. (2002) [23]	USA	WSW with BrC	Qualitative	Using a standardized methodology, focus groups were conducted with a convenience sample of WSW (*n* = 13) and heterosexual women (*n* = 28) diagnosed with BrC in the past 5 years. Thematic analysis and representative case study methods were used.	WSW reported higher stress associated with diagnosis, lower satisfaction with care received from physicians, and a trend toward lower satisfaction with available emotional supports. Overall QOL did not differ between groups.	Strengths: Multiple independent coders; standardized focus group methods with experienced moderator. Limitations: Small sample size.
Wheldon, Roberts, & Boehmer (2019) [27]	USA	Female BrC survivors stage 0-III (*n* = 330 lesbian, *n* = 525 heterosexual)	Quantitative	Tested a theoretical framework to explain differences in coping between lesbian and heterosexual BrC survivors; five subscales from the Mini-MAC Scale used to measure coping with BrC among women post-treatment; mediation analysis used to examine the explanatory power of life course factors (e.g., parenting and education) in explicating the association between sexual identity and coping responses.	Lesbian women had less avoidant coping strategies and lower levels of anxious preoccupation than heterosexual counterparts.	Strengths: Replicates other literature indicating resilience among lesbian BrC survivors. Limitations: Cross-sectional study with a non-random sample that is mostly white.
White & Boehmer (2012) [24]	USA	Partnered WSW (*n* = 15) diagnosed with nonmetastatic BrC from 2000 to 2005	Qualitative	One-on-one semi-structured telephone interviews were recorded, transcribed, and analyzed to identify emergent themes.	Themes describing WSW survivors' perceptions of support included female partners as the singular and most valuable source of support; sense of support from partner due to conversations about survivor distress; perceived partner distress; increased partner burden; and sense of support from partner sharing in a life beyond cancer.	Limitations: Mostly White sample; small sample size.
***Colorectal cancer (CRC)***						
Baughman et al. (2017) [109]	USA	Queer survivors with a diagnosis of stage III CRC (*n* = 8)	Qualitative	Semi-structured telephone interviews	Participants reported economic challenges associated with insurance coverage, employment, and housing as well as social isolation.	Strengths: This is the only known study focusing on queer CRC survivors; Sample was diverse in sex, sexual orientation, and socioeconomic status. Limitations: Lack of racial/ethnic diversity in sample; lack of staging in CRC respondents.
Boehmer et al. (2020a) [110]	USA	Colorectal cancer survivors with stage I-III disease from four state cancer registries (California, Seattle-Puget Sound, Georgia, and Florida) (*n* = 480)	Quantitative	Survivors were mailed a questionnaire which asked about sexual orientation. Respondents participated in a telephone interview assessing quality of cancer care (e.g., physician communication, nursing care, and coordination of care). General linear models and logistic regression was used to obtain models that best fit each quality of care measure.	There were no statistically significant differences between sexual minority cancer survivors (*n* = 127) and heterosexual survivor (*n* = 353) ratings of physician communication, nursing care, and coordination of care; however sexual minorities rated quality of care as excellent more often than heterosexual survivors.	Strengths: Population-based state registry recruitment; rigorous quantitative methods. Limitations: Primarily White sample; reports did not differentiate between male and female sexual minority survivors.
***Prostate cancer (PrC)***						
Allensworth-Davies et al. (2016) [111]	USA	Gay men age 50+ with a diagnosis of PrC (*n* = 111)	Quantitative	Cross-sectional national survey using multivariate generalized linear modeling with primary outcome as masculine self-esteem.	Men who were comfortable disclosing their sexual orientation to their doctor had higher masculine self-esteem scores. Mental health was positively correlated with masculine self-esteem. This study distinguished experiences of gay PrC survivors from heterosexual counterparts in terms of stigma and resilience.	Strengths: use of several validated scales (SF-12, EIPC, PDRQ-9); control of confounding variables; diversity of the study population in terms of age, insurance type, employment status, and treatment protocol. Limitations: convenience sample; lack of racial/ethnic diversity of participants.
Jägervall et al. (2019) [34]	Sweden	Gay men with a diagnosis of PrC (*n* = 11)	Qualitative	Participants were recruited through purposive sampling through SGM-identified networks and participant referral of other eligible men. Semi-structure interviews of 30–90 min were conducted.	Participants reported loss of ejaculate, erectile dysfunction, weaker orgasms, and penetration difficulties. These challenges were sometimes associated with feelings of loss, unattractiveness, and disability.	Strength: First study in Sweden exploring gay men’s experiences of sexual changes. Limitations: Small and non-diverse sample.
Capistrant et al. (2016) [41]	USA	Gay and bisexual men who had been diagnosed with PrC recruited from a national cancer support group network (*n* = 30)	Qualitative	One-on-one interviews probed for experiences with providers; health; sexual functioning; relationships; and informational, instrumental, and emotional support throughout PrC.	Single men in the study reported a need for independence; partnered men indicated varying levels of dependence on partners for support; many participants wished for more support options tailored for gay and bisexual men. In contrast to literature describing heterosexual PrC survivors, most support for gay and bisexual men came from family and friends rather than partners.	Strengths: One of few studies of gay and bisexual PrC survivors. Limitations: The sample was not very diverse: almost all participants were White, gay, and HIV-. There was not clear theoretical basis for the analysis.
Crangle, Latini, & Hart (2017) [35]	USA and Canada	MSM who had been diagnosed with PrC within the last 4 years (*n* = 92)	Quantitative	Convenience sample of MSM recruited through a variety of methods; demographic, medical information, and measures of attachment and illness intrusiveness were collected; mediation models were tested using bootstrapping to examine each attachment dimension on subscales of IIRS, controlling for age and days since diagnosis.	Younger age and greater anxious attachment were associated with greater illness intrusiveness. Greater anxious attachment was associated with less comfort with outness. Less comfort with being out to one’s provider mediated the association between greater anxious attachment and more illness intrusiveness. This means that comfort with outness could reduce illness intrusiveness for MSM with anxious attachment styles.	Strengths: use of previously developed scales (RQ; IIRS) and a newly developed Outness Inventory that demonstrated strong reliability (subscales α≥.86). Limitations: Cross-sectional design; self-report data; predominantly White, highly educated sample; variable internal reliability of the RQ.
Hart et al. (2014) [96]	USA and Canada	SM who had been diagnosed with PrC within the last 4 years (*n* = 92)	Quantitative	Convenience sample of MSM recruited through a variety of methods; demographic, medical information, and measures of QOL, HRWOL, change in sexual activity, sexual side effects, satisfaction with care, self-efficacy for symptom management, disease-specific anxiety, illness intrusiveness, and “outness level” collected; mean scores were calculated and compared to published population means in studies using the same scale, where possible; open-text responses reported descriptively.	MSM reported significantly worse urinary and bowel function, greater bother of lack of ejaculation than heterosexual peers from other published studies, lower satisfaction with PrC care—but overall health status was similar. MSM reported significantly worse mental but not worse physical health functioning than heterosexual peers. Nearly half (49%) of MSM reported changes to erectile function and 40.2% indicated less frequency of sexual activity. MSM reported painful erections, climacturia, low libido, changes in self-image, partner struggling with relationship changes, and significant changes in sexual experiences due to lack of ejaculation.	Strengths: Use of validated scales (EPIC; SF-36; MSHQ; CapSURE; ILLS) and a newly developed Outness Inventory that demonstrated strong reliability. Limitations: Predominantly White, educated, and “out” self-selected sample; cross-sectional design; variation in study design of comparison groups.
Hartman et al. (2013) [45]	Canada	Homosexual couples following one partner’s radical prostatectomy due to PrC (*n* = 6; i.e., three couples)	Qualitative	Interpretative phenomenological analysis using inductive coding.	Major themes included acknowledging, accommodating, and accepting sexual changes. Unlike research on heterosexuals, the role of open relationships was helpful in 2 of the 3 partners studied. These couples also benefited from communication (similar to heterosexual couples). For the third couple, sexual dysfunction was so significant that communication did not feel beneficial in helping with sexual health.	Strengths: This study provides a counternarrative to the dominant heterosexual assumptions about sexual health following radical prostatectomy. Limitations: The study was exploratory with a small sample.
Lee, Breau, & Eapen (2013) [43]	Canada	MSM with PrC (*n* = 15)	Quantitative	Pilot study comparing post-treatment QOL in MSM who had surgery to MSM who had radiation for treatment of PrC.	While the sample size precluded statistical comparisons, the radiation group appeared to have fewer sexual side effects post-treatment in terms of retained ability for penetrative and receptive intercourse.	Strengths: Use of validated scales (EPIC, MSHQ). Limitations: Pilot study with small sample prevented statistical analysis; researcher-created sexual function survey not validated.
Lee et al. (2015) [112]	Canada	MSM with PrC (*n* = 16)	Qualitative	MSM were interviewed face-to-face or via video conferencing and asked about sexual QOL after PrC. Interviews were recorded, transcribed, and analyzed.	Themes from semi-structured interviews included sexual dysfunction (e.g., erectile, urinary, ejaculation, and orgasmic), intimacy challenges, and lack of support for cancer and psychosocial needs. Sexual QOL and relationship confidence were lower for those with greater sexual dysfunction. Coping was challenged by lack of support.	Strengths: The first qualitative study exploring the impact of PrC on MSM survivors’ sexual experiences; rich data to develop a new QOL instrument specific to MSM PrC survivors. Limitations: Sociodemographic diversity not discussed.
Hoyt et al. (2020) [113]	USA	Gay men who had been diagnosed with PrC (*n* = 11)	Qualitative	Focus groups (*n* = 3) with gay PrC survivors (*n* = 11) using conventional content analysis.	Major challenges for participants included minority stress, intimacy/sexuality concerns, impact on life outlook, healthcare experiences, social support and the gay community, and intersectional identities.	Strengths: 2-3-hour time for focus groups allowed for participant directed discussion; racial diversity in sample. Limitations: Small sample size.
McConkey & Holborn (2018) [44]	Ireland	Gay men with PrC (*n* = 8)	Qualitative	In-depth interviews based on phenomenology were conducted with gay PrC survivors; interviews were recorded and transcribed; data was divided into “meaning units”; credibility and trustworthiness were bolstered by reflexivity, memoing, field notes of interviewee behaviors, and peer review of thematic descriptions from the data.	Three major themes that emerged included: (1) the experience of diagnosis and treatment, marked by shock at diagnosis, overwhelm during decision-making, sexual impacts of treatment; and degree of access to a nurse specialist; (2) experiences of health care service, including disclosure and communication with the care team; and (3) sources of support (e.g., family, friends), heteronormativity of support groups, and lack of gay community resources.	Strengths: First-known study to explore gay PrC survivor experiences in Ireland. Limitations: Lack of racial, national, and educational diversity in sample (important since 14-23% of the gay population in Ireland is foreign born).
Motofei et al. (2011) [39]	Romania	Romanian PrC survivors (*n* = 17 heterosexual men, *n* = 12 gay men)	Quantitative	Gay and heterosexual PrC survivors were asked about sexual functioning prior to and after starting bicalutamide monotherapy. A 2 × 2 factorial ANOVA compared heterosexual v. gay and pre- v. post-exposure to bicalutamide.	Mean IIEF scores were lower after bicalutamide exposure for the full group (*p* < .001) with greater reductions in scores for gay v. heterosexual survivors after exposure.	Strength: First-known study of gay PrC cancer survivors in Romania; only study found to examine sexual impact of a drug by sexual orientation; use of validated scale (IIEF). Limitations: Small sample size; potential for recall bias; binary design does not account for bisexuality.
Polter et al. (2019) [38]	USA	PrC survivors who participated in the RESTORE study (*n* = 191) including HIV+ (*n* = 24) and HIV− (*n* = 167) MSM	Quantitative	Cross-sectional, online survey of MSM treated for PrC examined sexual function, bother, and HRQOL using MANOVA and multivariate linear regression to evaluate association of HIV status and HRQOL after controlling for demographic and sexual characteristics.	HIV+ status was associated with lower mean urinary, sexual, and bowel scores on the EPIC after controlling for demographic and sexual characteristics. HRQOL did not differ by HIV status.	Strengths: Use of validated scales (EPIC, SF-12). Limitations: Small number of HIV+ men in the sample; cross-sectional design; evidence of fraudulent responses (procedure used to omit 200 responses was not described).
Rosser et al. (2016) [42]	USA	Gay and bisexual men who had been diagnosed with PrC (*n* = 19)	Qualitative	In-depth telephone interviews with gay and bisexual men who had radical prostatectomies.	Themes included shock at diagnosis; depression; anxiety, grief, loss of sexual confidence; changes in sense of “maleness,” gay/bisexual identity, sex-role identity; sex interest and partners; disclosure of cancer survivorship status; and changes to relationships including renegotiation of exclusivity with partners.	Strengths: One of few studies focused on gay and bisexual PrC survivors. Limitations: Small sample size.
Rosser et al. (2017) [40]	USA and Canada	Gay and bisexual men who had been diagnosed with PrC (*n* = 193)	Quantitative	Online survey regarding sexual functioning measured using the Expanded Prostate Cancer Index Composite (EPIC) and a tailored Gay Sexual Functioning Inventory (GSFI).	MWM had worse urinary and hormonal function, but better sexual function than published norms. Most participants described their sexual functioning as fair to poor following treatment with less than a quarter of men reporting sufficient erections for insertive anal sex. Anal receptive men reported pain during sex after treatment. Over half of respondents reported urination problems during sex. Sexual functioning significantly predicted long-term positive health outcomes.	Strengths: Large sample. Limitations: Convenience sample; predominantly White.
Rosser et al. (2019) [114]	USA and Canada	MSM who had been diagnosed with PrC (*n* = 193)	Quantitative	Survey regarding rehabilitation treatments for sexual and urinary effects of PrC treatment—what they were offered, what they tried, and what their satisfaction was with the outcomes.	The most common problems reported were loss of ejaculate (93.8%), erectile difficulties (89.6%), change in sense of orgasm (87.0%), loss of sexual confidence (76.7%), changes to the penis (65.8%), increased pain in receptive anal sex (64.8%), urinary incontinence not related to sex (64.2%) and urinary incontinence during sex (49.2%). Of these factors only loss of ejaculate, erectile difficulties and nonsexual urinary problems were commonly discussed by clinicians during PrC treatment. Satisfaction with specific rehabilitation options varied widely.	Strengths: First-known study to investigate rehabilitation options to address sexual and urinary effects of PrC treatment. Large sample size. Limitations: Mostly White MSM. Cross-sectional.
Thomas et al. (2013) [47]	Australia	Australian MSM with a PrC diagnosis within the last 7 years (*n* = 10)	Qualitative	An asynchronous, online focus group was hosted over 4 weeks with MSM PrC survivors discussing impact of PrC on their lives.	Respondents mentioned accessing support, the challenges of incontinence and sexual changes, changes to sexual relationships, and divergent emotional responses (resilience v. negative outcomes). Respondents also indicated that general practitioners were more empathic than their urologists, and felt their emotional needs were not adequately addressed and that interactions with urologists were often distressing.	Strengths: Leveraging online technology to conduct qualitative work is innovative. Limitations: Recall and self-selection bias; all-White sample prevents exploration of diverse MSM outcomes.
Thomas et al. (2018) [115]	Australia	Australian PrC survivors (*n* = 813)	Quantitative	An online survey asked respondents about demographics, treatment modality for PrC, body image, self-esteem, sexual function and urinary function; a 2 × 2 ANCOVA was conducted to examine the main effect of two factors: sexual orientation and PrC diagnosis over six outcomes: self-esteem, urinary function, sexual function, appearance evaluation, health evaluation, and health orientation; differences in age and Gleason score were also examined.	Never-diagnosed respondents were statistically significantly younger than cancer survivors. Overall, gay respondents had statistically significantly higher age-adjusted self-esteem scores compared to heterosexual peers. PrC survivors had statistically significantly worse urinary and sexual function and health orientation than never- diagnosed peers. No statistically significant differences in outcomes were found between gay and heterosexual PrC survivors, although urinary function differences only narrowly failed to meet statistical significance (*p* = .054).	Strengths: Use of validated measures (EPIC, MBSRQ). Limitations: Cross-sectional design; small sample size of gay men with PrC; potential for self-selection (via social media recruitment) and self-report bias.
Torbit et al. (2015) [93]	USA and Canada	MSM who received a PrC diagnosis within the prior 4 years (*n* = 92)	Quantitative	A multiple mediation design was used to test both self-efficacy and satisfaction with care on the relationship between physical symptom severity and FOR for PrC survivors.	Worse physical symptoms were associated with greater FOR. Self-efficacy and satisfaction of care mediated the statistically significant relationship between worse bowl function, worse hormone function, and worse sexual function with FOR, respectively. Self-efficacy and satisfaction did not mediate worse urinary function and FOR, but did explain 61% of the variance in the sample for that outcome.	Strengths: Use of a validated tool (EPIC) and tools from prior studies to measure self-efficacy and satisfaction with care. Limitations: Mostly White, educated, partnered sample; cross-sectional design; self-report data.
Ussher et al. (2016) [94]	Australia	Australian PrC survivors (*n* = 124 MSM, *n* = 225 heterosexual men)	Quantitative	Participants were recruited through urology and primary care practices, support groups, SGM community groups, social media, and cancer research volunteer databases; multiple regression and independent samples t-tests assessed group differences; Pearson’s correlations assessed associations between MSM and heterosexual samples; multiple linear regression was used to identify meaningful predictor variables for HRQOL.	MSM were younger, less likely to be partnered, and more likely to have casual sex than heterosexual peers in the sample. MSM reported worse HRQOL, worse masculine self-esteem, lower satisfaction with care, higher psychological and cancer-related distress, greater ejaculation concerns, higher sexual functioning, and more sexual confidence at statistically significant levels compared to heterosexual peers.	Strengths: Use of validated tools (FACT-P, BSI-18, CSFQ-M, DSC, EPIC, MAX-PC, PrCQOL). Limitations: Differences between MSM and heterosexual samples (e.g., age, ethnicity, employment status, relationship status, and treatments received).
Ussher et al. (2017) [36]	Australia, New Zealand, UK, USA	Australian MSM PrC survivors (*n* = 124) and their partners (*n* = 21); subset interviewed (*n* = 46 survivors, *n* = 7 partners)	Mixed Methods	An online survey of MSM PrC survivors (*n* = 124) and their male partners (*n* = 21) explored sexual experiences, relationships, and psychological wellbeing after treatment; a subset of this sample opted to also be interviewed (*n* = 46 survivors and *n* = 7 partners); descriptive statistics from the survey and themes from the interviews were reported.	Survivors reported erectile dysfunction, emotional distress, feelings of sexual disqualification, both negative and adaptive impacts on gay identity, loss of libido, climacturia, pain during anal sex, lack of ejaculation, and penile shortening.	Strengths: Use of validated measures (EPIC-Sexual Domain, CSFQ-M, FACT-P); mixed methods design.
Wassersug et al. (2013) [37]	International: Primarily USA, Australia, Canada, and UK	Men (*n* = 556) from 17 countries with a diagnosis of PrC (*n* = 460 heterosexual men and *n* = 96 MSM)	Quantitative	Logistic regression and Wald tests assessed outcomes including sexual health, urinary incontinence, and depression.	No between-group differences were found for urinary incontinence or erectile dysfunction; however, MSM were more bothered by sexual impacts of PrC than heterosexual peers.	Strengths: International reach, adaptation of validated scale (EPIC). Limitations: Sample is largely affluent with access to the internet.
Wright et al. (2019) [116]	USA and Canada	MSM (*n* = 189) with a diagnosis of PrC recruited from Malecare, an online cancer support organization	Quantitative	Linear regression was used to compare participants with cats only, dogs only, both cats and dogs, or no pets on SF-12 mental and physical component scores.	Participants with pets had lower mental health scores than non-pet owners. Cat owners had better physical health than other groups.	Strengths: First study to look at companion animal ownership association with mental and physical wellbeing; use of validated scale (SF-12). Limitations: Convenience sample; cross-sectional design; inability to determine directionality of association; no heterosexual control group.
***Various cancers***						
Anderson et al. (2020) [46]	USA	Gay, older long-term AIDS survivor with moderate-to-severe demoralization and serious medical illness (e.g., cancer) (*n* = 18)	Quantitative	Participants were recruited for a single-arm open-label study of psilocybin-supported group therapy with 8–10 therapy visits and one psilocybin administration visit. Two-way repeated ANOVA was conducted at baseline, end-of-treatment and three-months post-treatment to assess mean demoralization using the Demoralization Scale II.	Clinically meaningful change in demoralization from baseline to 3-month follow up and no serious adverse events suggested that psilocybin-assisted group therapy could be an effective intervention for older long-term survivors of AIDS living with cancer or other serious medical illness to reduce demoralization.	Strengths: Use of validated scale (Demoralization Scale (II); focus on high-need, elderly population with serious medical illness; one of few interventions in the literature. Limitations: Feasibility study; larger study is needed to confirm results.
Boehmer et al. (2011) [31]	USA (California)	CHIS respondents ages 18-70 (*n* = 122,345 CHIS respondents, *n* = 10,942 survivors)	Quantitative	Pooled data from CHIS 2001, 2003, 2005 using logistic regression; primary outcomes were prevalence of cancer and self-reported health.*	WSW had ≥2.0 odds of fair/poor health compared to heterosexual counterparts with greater risk for racial minorities and older women; greater prevalence and younger diagnosis of cancer were reported by MSM compared to heterosexual counterparts but self-reported health was not different for MSM.	Strengths: Large, population-based sample (CHIS); first-known study to report prevalence of cancer and self-reported health of cancer survivors by sexual orientation. Limitations: Data collected only from one state; self-reported nature of the data.
Boehmer et al. (2019) [30]	USA	BRFSS respondents who had a past diagnosis of cancer (*n* = 68,593 heterosexual women, *n* = 1,931 WSW)	Quantitative	Secondary data analysis of 2014-2017 years of BRFSS data. Survivors were categorized with an access deficit if any one of the following were true: no health insurance, delaying care, avoiding care due to cost, and lacking a trusted physician. Weighted analysis computed odds ratios and 95% confidence intervals using cumulative logit models and logistic regression, taking into account confounders.	WSW reported more access to care deficits—including lack of health care coverage, having no personal physician, avoiding care due to cost, and being without an annual visit—compared to heterosexual peers (*p* < .0001). WSW with deficits had poorer physical and mental QOL and trouble concentrating compared to heterosexual peers.	Strengths: Use of a large, population-based sample. Limitations: Small WSW sample sizes prevented subanalyses.
Boehmer et al. (2020b) [48]	USA	Cancer survivors from the Behavioral Risk Factor Surveillance System (2014–2018)	Quantitative	Multiple logistic regression was used to estimate cancer prevalence and odds ratios for a variety of health outcomes and behaviors for 954,800 cisgender and transgender individuals with a history of cancer. The sample included 1877 transgender women, 1344 transgender men, 876 gender nonconforming individuals, 410,422 cisgender men and 540,389 cisgender women.	Transgender men had a higher rate of cancer compared to cisgender men, but not cisgender women. No other prevalence differences were found. Gender nonconforming survivors reported greater physical inactivity, alcohol use and depression compared with cisgender peers. Transgender men reported poorer physical health and more comorbidities than cisgender men or women. Transgender women reported more diabetes than cisgender men or women and greater cardiovascular disease compared to cisgender women.	Strengths: Population-based study; one of very few studies focused on transgender and gender nonconforming population. Limitations: No notable limitations
Bryson et al. (2018) [49]	Canada	SGM breast and gynecological cancer survivors (*n* = 81)	Qualitative	Purposive sampling used to recruit diverse sample of SGM BrC survivors across Canada; semi-structured interviews conducted to explore patient experiences of care, health outcomes and decision-making.	This study reported on perceptions of how intersectional identity influenced feelings of safety and interactions with health care providers. It provides evidence that cisnormative systems negatively shaped care experiences for genderqueer people. Relevant to the present review outcomes reported were: physical impacts of cancer treatment that resulted in altered experiences of gender in society; lack of preparation or hormonal treatment for surgery-induced menopause; and mental health effects associated with lack of hormonal treatment.	Strengths: Diversity of sample; rich exploration of narratives; large sample; one of two known studies of genderqueer cancer survivors sharing their experiences in their own words. Limitations: No notable limitations.
Hutchcraft et al. (2020) [97]	USA	Lesbian and bisexual women who participated in the National Health Interview Survey 2013–2018	Quantitative	Weighted, multivariable logistic regression was used to estimate odds ratios of heterosexual, lesbian, and bisexual women with a history of cancer on health-related QOL and health behaviors.	With heterosexual women as the reference group, lesbian women were 58% more likely to self-report fair/poor health, almost twice as likely to report chronic obstructive pulmonary disease or heart conditions, and twice as likely to be current smokers; bisexual women reported three times the rate of psychological distress, twice the rate of heart conditions, nearly three times the rate of food insecurity, and were less likely to have a recent mammogram.	Strengths: National population-based sample, rigorous methods. Limitations: No notable limitations.
Jabson, Farmer, & Bowen (2015) [91]	USA	Cancer survivors participating in the NHANES from 2001 to 2010 (*n* = 576 heterosexual women, *n* = 26 WSW).	Quantitative	NHANES data from 2001 to 2010 were pooled and 602 cancer survivors were identified. Between-group (WSW v. heterosexual) characteristics, health behaviors, and self-reported health were compared using chi-square and t-tests; logistic regression was used to compare WSW v. heterosexual aORs; propensity score adjustment used for sociodemographic variables.	4.3% of the sample self-identified as WSW. WSW were 2.5 times more likely to report past illicit drug use and 60% less likely to report current health as good compared to heterosexual peers.	Strengths: Population-based sample. Limitations: Small sample of sexual minority cancer survivors in NHANES data due to lack of data collection of sexual orientation from 2001 to 2006 limited the power of the study.
Kamen et al. (2014) [117]	USA	Men who reported sexual orientation in the BRFSS in 2009 from Arizona, California, Massachusetts, Ohio, and Wisconsin (*n* = 14,354)	Quantitative	The complex sampling procedure in SPSS (v. 20.0) weighted the sample based on demographic variables and state of residence; statistically significant between-group differences were used as co-variates for a logistic regression and t-tests examining outcomes.*	Gay men were 82% more likely to report a cancer diagnosis (*p* < .05) and were more likely to report less exercise, more distress, and greater alcohol and/or tobacco use. These health behaviors were shown to continue after a cancer diagnosis for gay men.	Strengths: Population-based sample from five states; first study to examine cancer disparities among gay men. Limitations: Cross-sectional design of BRFSS; potential lack of disclosure of sexual orientation among respondents.
Kamen et al. (2015) [92]	USA	LiveStrong survey respondents: *n* = 207 SGM, *n* = 4899 heterosexual cancer survivors in 2010	Quantitative	Propensity matched cancer survivors (*n* = 621 heterosexual v. 207 LGBT survivors) assessed for distressed, difficulties with social relationships, fatigue and energy; symptoms assessed through dichotomous yes/no items and analyzed using Poisson regression; subgroup analyses by sex conducted.	SGM men reported greater depression and more relationship difficulties compared to heterosexual counterparts. SGM women did not have differences compared to heterosexual peers.	Strengths: First-known study to examine psychological distress of sexual minority cancer survivors. Limitations: Cross-sectional design of the study.
Kamen et al. (2015) [50]	USA	291 SGM cancer survivors (*n* = 159 MSM, *n* = 123 WSW, *n* = 7 transgender men, *n* = 2 transgender women)	Quantitative	Participant demographics, cancer diagnosis, experiences of care, support-related factors, and self-rated health were assessed through a researcher-developed survey; descriptive data reported; logistic regression used to compare outcomes.	Parental support was the strongest single factor associated with good health followed by having a partner present during cancer diagnosis.	Strength: One of the largest studies of SGM cancer survivors at the time of publication. Limitations: Researcher-created survey that has not been validated; self-report data; cross-sectional design; self-selection bias; recall bias of support and comparison of support at diagnosis with present self-reported health.
Kamen et al. (2016) [95]	USA	Queer (*n* = 10) and heterosexual (*n* = 12) cancer survivors	Quantitative	Randomized controlled trial of a 6-week exercise intervention comparing survivor-only v. survivor-caregiver dyad using independent samples t-tests.	At baseline, queer survivors reported greater depression (*p* = .01) and fewer steps walked (*p* = .03) compared to heterosexual counterparts. Post-intervention, there were no differences between queer v. heterosexual survivors, but survivors with partner support had a significantly greater reduction in depressive symptoms compared to the survivor-only group.	Strengths: One of very few interventional studies to improve QOL of queer cancer survivors; use of validated scales (CES-D, STAI, DSQ). Limitations: Small sample size.
Lisy et al. (2019) [51]	Australia	Australian cancer survivors (*n* = 2115)	Quantitative	Cancer survivors diagnosed between 2009 and 2013 were identified through the Victorian Cancer Registry and asked to complete a survey about demographics, QOL, social difficulties, and information needs; descriptive data reported as well as between-group differences (SGM v. heterosexual).	Of the 2115 Australian cancer survivors who responded to the survey, 33 (1.6%) disclosed SGM status. SGM survivors had significantly fewer financial, support, and communication challenges post-treatment but greater challenges with diet and lifestyle than heterosexual peers. SGM survivors were more likely to report anxiety/ depression and body image challenges, but not at a statistically significant level.	Strengths: First population-based survey of SGM cancer survivors in Australia; use of some (unspecified) validated measures. Limitations: Questionnaires were not validated; small SGM sample; sexual orientation was not decoupled from gender identity.
Matthews et al. (2016) [118]	USA	SGM cancer survivors (*n* = 175)	Quantitative	Cancer survivors were recruited through SGM-serving organizations to take an 82-item online survey asking about demographics, cancer type, comorbid conditions, health behaviors, and QOL; descriptive statistics summarized demographics; multivariable models were created to explore associations with physical and mental subscales of the SF-12.	Lower physical QOL scores were associated with older age at diagnosis, breast or gynecological cancer, medical comorbidities, overweight or obesity, and cancer recurrence (*p* < .05). Lower mental QOL scores were associated with younger age at diagnosis, lack of physical activity, FOR, lower levels of social and emotional support, and participation in therapy/support groups (*p* < .05).	Strengths: Use of a validated QOL measure (SF-12); diversity of type and stage of cancer as well as geography distribution across USA Limitations: Cross-sectional, convenience sample; limited racial/ethnic diversity in sample; no comparison group.
Seay et al. (2018) [119]	USA	SGM cancer survivors (*n* = 114)	Quantitative	LGBT cancer survivors were recruited through National LGBT Cancer Project for a survey capturing demographics, social support, posttraumatic stress, and cancer survivorship care needs.	Most respondents reported unmet needs (73%) and over half reported unmet psychological and sexual care needs. Those who reported that providers were not culturally competent had greater unmet needs (*p* = .0.01 and greater posttraumatic stress (*p* = 0.035).	Strengths: Community-based participatory approach to study design; use of validated surveys (SCNS-SF34, IES-6, MOS-SS). Limitations: Cross-sectional, limited participation by cisgender women and gender diverse persons, limited racial/ethnic diversity in sample.

*Abbreviations*: *aOR* adjusted odds ratio, *DCIS* ductal carcinoma in situ, *BrC* breast cancer, *BRFSS* Behavioral Risk Factor Surveillance System, *CRC* colorectal cancer, *FOR* fear of recurrence, *PrC* prostate cancer, *HRQOL* health-related quality of life, *MSM* men who have sex with men, *SGM* sexual and gender minorities, *QOL* Quality of Life, *WSW* sexual minority women

*Appropriate tests were conducted to compare demographic and clinical characteristics between groups

## Abbreviations

AYA: Adolescents and young adults
BMI: Body mass index
HIV: Human immunodeficiency virus
LGBTQI: Lesbian, gay, bisexual, transgender, queer, and/or intersex
MSM: Men who have sex with men
NIH: National Institutes of Health
PICO: Patient/Population/Problem, Intervention, Comparison, Outcome
SGM: Sexual and gender minorities
QOL: Quality of life
WSW: Women who have sex with women
